# Mortality and pre‐hospitalization use of low‐dose aspirin in COVID‐19 patients with coronary artery disease

**DOI:** 10.1111/jcmm.16198

**Published:** 2020-12-18

**Authors:** Shuai Yuan, Peng Chen, Huaping Li, Chen Chen, Feng Wang, Dao Wen Wang

**Affiliations:** ^1^ Division of Cardiology Department of Internal Medicine Tongji Hospital Tongji Medical College Huazhong University of Science and Technology Wuhan China; ^2^ Hubei Key Laboratory of Genetics and Molecular Mechanisms of Cardiological Disorders Wuhan China

**Keywords:** aspirin, coronary artery disease, COVID‐19, SARS‐CoV‐2

## Abstract

To determine whether pre‐hospitalization use of aspirin is associated with all‐cause mortality in coronavirus disease 2019 (COVID‐19) patients with coronary artery disease (CAD). We recruited 183 adult patients with CAD diagnosed with COVID‐19, including 52 taking low‐dose aspirin (mean [SD] age, 69.7 [1.1] years; 59.6% men) and 131 without using aspirin (mean [SD] age, 71.8 [0.9] years; 51.9% men), who were admitted in the Tongji hospital in Wuhan, China from January 10, 2020 to March 30, 2020. There was no difference on in‐hospital mortality between aspirin group and non‐aspirin group (21.2% vs. 22.1%, *P* = .885). Similarly, for critically severe COVID‐19 patients, the mortality in aspirin group was close to that in non‐aspirin group (44% vs. 45.9%, *P* = .872). Moreover, the percentage of patients with CAD taking low‐dose aspirin did not differ between those survivors and non‐survivors (28.7% vs. 27.5%, *P* = .885). Meanwhile, the usage of aspirin was not correlated with all‐cause mortality in multivariate analysis (OR = 0.944, 95% CI: 0.411‐2.172, *P* = .893). Collectively, our study suggested that the pre‐hospitalization use of low‐dose aspirin was not associated with the clinical outcome of patients with CAD hospitalized with COVID‐19 infections.

**Abbreviations**
ACC: American College of Cardiology
AHA: American Heart Association
ALT: Alanine transaminase
APTT: Activated partial thromboplastin time
ARDS: Acute respiratory distress syndrome
AST: Aspartate transaminase
BUN: Blood urea nitrogen
CAD: Coronary artery diseases
CI: Confidence interval
CK: Creatine kinase
CKD: Chronic kidney disease
CK‐MB: Creatine kinase‐MB
COPD: Chronic obstructive pulmonary disease
COVID‐19: Coronavirus disease 2019
Cr: Creatinine
CVD: Cardiovascular disease
DBP: Diastolic blood pressure
DIC: Disseminated intravascular coagulation
Hb: Haemoglobin
hs‐CRP: High sensitive C reaction protein
hs‐cTnI: High sensitive cardiac troponin I
IL‐1β: Interleukin 1β
IL‐2R: Interleukin 2 receptor
IL‐6: Interleukin 6
IL‐8: Interleukin 8
IQR: Interquartile range
LDH: Lactate dehydrogenase
LDL‐c: Low‐density lipoprotein cholesterol
Lymph: Lymphocyte
Mb: Myoglobin
MERS: Middle East Respiratory Syndrome
Neut: Neutrophil
NSAIDs: Non‐steroidal anti‐inflammatory drugs
NT‐proBNP: N‐Terminal pro‐brain natriuretic peptide
OR: Odds ratio
PLT: Platelet
PT: Prothrombin time
RBC: Red blood cell
SaO2: Oxyhaemoglobin saturation
SARS: Severe acute respiratory syndrome
SBP: Systolic blood pressure
TBIL: Total bilirubin
TC: Total cholesterol
TNF‐α: Tumour necrosis factor‐α
WBC: White blood cell
WHO: World Health Organization

## 1 INTRODUCTION

In December 2019, the coronavirus disease 2019 (COVID‐19), caused by an infection with a novel coronavirus, officially named severe acute respiratory syndrome coronavirus 2 (SARS‐CoV‐2), was first reported in Wuhan, Hubei province, China.^1^ Emerging data suggest that patients with severe COVID‐19 have a relative high incidence of coronary artery disease (CAD) and CAD may be associated with an high risk of mortality due to COVID‐19 among patients admitted to hospital.^2^, ^3^, ^4^

Aspirin is a typical non‐steroidal anti‐inflammatory drug with strong anti‐inflammatory, anti‐thrombotic and analgesic pharmacological effects. Long‐term low‐dose aspirin (75‐150 mg daily) can effectively prevent the incidence of ischaemic cardiovascular and cerebrovascular events.^5^, ^6^ However, prophylactic use of low‐dose aspirin is currently controversial in patients with COVID‐19. The Belgian Federal Agency for Medicines and Health Products released a statement on March 16, 2020 stating that NSAIDs can lead to serious complications. Equally, a report by French Authorities suggested the use of ibuprofen in COVID‐19 patients was detrimental to patient's condition and recovery.^7^ Previous experiments suggested that NSAIDs may increase the expression of ACE2 in rats,^8^ a pivotal receptor of SARS‐CoV‐2, which potentially increasing SARS‐CoV‐2 infectivity.

As aspirin irreversibly inhibits platelet cyclooxygenase, and even causes thrombocytopenia in a percentage of patients,^9^ there are concerns that use of aspirin significantly increases the risk of bleeding. Besides, NSAIDs had been shown to alter the function of neutrophils, delaying inflammation and may accelerate the progression of pneumonia.^10^ In fact, the side effects of NSAIDs on COVID‐19 are largely attribute to its anti‐inflammatory effects. Paul Little, professor of primary care research at the University of Southampton suggested that patients on low‐dose aspirin for secondary prevention of cardiovascular disease should be advised to continue, noting that aspirin has anti‐inflammatory effects only at much higher doses.^11^ A recent study showed there was no difference of aspirin usage between the survival and non‐survival of COVID‐19 patients.^12^ To our knowledge, no study reported the relationship between low‐dose aspirin and COVID‐19 patients with CAD. As an important drug for secondary prevention of coronary artery disease, it is important to explore the relationship between the baseline use of low‐dose aspirin and the clinical prognosis of COVID‐19 patients.

## 2 MATERIALS AND METHODS

### 2.1 Study design and participants

Patients with COVID‐19 admitted to the Tongji Hospital of Wuhan (Hubei Province, China) from January 10, 2020, to March 30, 2020, were included in this retrospective analysis. The study was approved by the Ethics Committee of Tongji Hospital and Tongji Medical College. The requirement for written informed consent was waived by the Ethics Committee because of the retrospective and anonymous nature of the data.

COVID‐19 was diagnosed according to the Guidance for Corona Virus Disease 2019 (6th edition) released by the National Health Commission of China.^13^ The critically ill COVID‐19 patient was defined as meeting any one of the following items, according to the Diagnosis and Treatment Plan of COVID‐19 suggested by National Health Commission of China: Respiratory failure requiring mechanical ventilation; Shock; Patients combined with other organ failure needed ICU monitoring and treatment. Patients with coronary artery disease was defined by presenting more than one of the following conditions: 1) existing myocardial infarction; 2) treated with percutaneous coronary intervention or a coronary artery bypass graft; 3) > 50% stenosis of at least 1 of the 3 major coronary arteries (left anterior descending, circumflex, or right coronary artery) demonstrated by coronary angiography.

The inclusion criteria included COVID‐19 patients with pre‐existing coronary artery disease, aged over 18 years, who were admitted to Tongji Hospital from January 10, 2020 to March 30, 2020. The exclusion criteria included incomplete medical records, patients with coronary artery disease who received rivaroxaban or dabigatran but not aspirin. Based on their anti‐thrombotic drug treatments before hospitalization, the patient cohort was assigned into two groups: the low‐dose (75‐150 mg daily) aspirin treated group (aspirin, n = 52) and the non‐aspirin treated group (non‐aspirin, n = 131).

### 2.2 Data Collection

All clinical data were extracted from patients’ electronic medical records. Demographics, medical history, and coexisting comorbidities including CAD, COPD, CKD, diabetes, and hypertension were collected by the attending physician, and vital signs, laboratory findings were recorded on admission.

### 2.3 Statistical analysis

Normally and abnormally distributed continuous variables were represented by mean ± SD and median (interquartile range), respectively. Categorical variables were represented by number (percentage). Normally and abnormally distributed continuous variables were compared using the Student's t‐test and the Mann‐Whitney U test, respectively. Categorical variables were compared using the chi‐squared test or Fisher exact test. Categorical and consecutive variables were evaluated by logistic regression analysis for their ability to predict mortality. A 2‐sided α of less than 0.05 was considered statistically significant. Data were analysed using SPSS 21.0 for Windows (SPSS Inc, Chicago, IL, USA).

## 3 RESULTS

As of March 30, 2020, 2912 consecutive COVID‐19 patients were admitted to Tongji Hospital. According to inclusion and exclusion criteria, a total of 2886 patients were included in this retrospective study, including 2703 (93.7%) non‐CAD patients and 183 (6.3%) CAD patients. The mean age was 59.1 years and 1402 (49.1%) were men. Patients with CAD had increased risk of all‐cause‐mortality over non‐CAD patients (21.9% vs. 9.1%; *P* < .001). The characteristics and clinical outcomes of the patients with CAD compared with those without CAD were summarized in the Supplemental Table 1. Patients with CAD were older (71.2 vs. 58.3; *P* < .001) and had higher prevalence of comorbidities such as hypertension, diabetes, and chronic kidney disease. In addition, COVID‐19 patients with CAD were more seriously ill as they often had liver and kidney function damage and coagulation dysfunction, especially in myocardial damage compared with those without CAD.

**TABLE 1:** The characteristics and clinical outcomes of COVID‐19 patients with CAD

	COVID‐19 patients with CAD (n = 183)	COVID‐19 patients with CAD treated with aspirin (n = 52)	COVID‐19 patients with CAD treated without aspirin (n = 131)	*P* value
Demographics and clinical characteristics				
Age (y)	71.2 ± 0.7	69.7 ± 1.1	71.8 ± 0.9	0.15
Sex				0.345
Male (%)	99 (54.1)	31 (59.6)	68 (51.9)	
Female (%)	84 (45.9)	21 (40.4)	63 (48.1)	
Comorbidity				
Hypertension (%)	102 (55.7)	32 (61.5)	70 (53.4)	0.320
Diabetes (%)	40 (21.9)	13 (25.0)	27 (20.6)	0.517
COPD (%)	8 (4.4)	1 (1.9)	7 (5.3)	0.535
CKD (%)	18 (9.8)	6 (11.5)	12 (9.2)	0.626
Cerebrovascular disease (%)	9 (4.9)	3 (5.8)	6 (4.6)	1
Symptoms and signs				
Fever (%)	127 (69.4)	35 (67.3)	92 (70.2)	0.699
Cough (%)	105 (57.4)	34 (65.4)	71 (54.2)	0.168
Dyspnoea (%)	73 (39.9)	21 (40.4)	52 (39.7)	0.931
Temperature (℃)	36.5 (36.3‐37.0)	36.5 (36.2‐37.0)	36.5 (36.3‐37.0)	0.695
Respiratory rate (/min)	20.0 (20.0‐25.0)	20.0 (19.0‐22.0)	20.0 (20.0‐26.0)	0.072
Heart rate (bpm)	88.0 (78.0‐100.0)	88.0 (80.0‐96.8)	88.0 (78.0‐100.0)	0.981
DBP (mmHg)	80.0 (73.0‐88.0)	82.0 (70.0‐88.0)	80.0 (73.3‐88.0)	0.519
SBP (mmHg)	133.8 ± 1.4	135.2 ± 2.7	133.3 ± 1.7	0.583
Laboratory findings				
WBC (*10^9/L)	6.08 (4.99‐8.46)	6.61 (5.33‐8.63)	5.84 (4.87‐8.19)	0.391
RBC (*10^12/L)	4.01 ± 0.05	4.05 ± 0.77	4.00 ± 0.06	0.438
Neut (*10^9/L)	4.16 (3.05‐6.79)	4.61 (3.33‐7.07)	4.11 (2.99‐6.79)	0.497
Hb (g/L)	123.0 (110.0‐136.0)	125.5 (107.3‐134.0)	122.5 (110.0‐136.3)	0.770
Lymph (*10^9/L)	1.05 (0.68‐1.52)	1.15 (0.57‐1.60)	1.03 (0.69‐1.50)	0.823
PLT (*10^9/L)	209.0 (152.8‐285.0)	213.0 (153.8‐297.5)	207.5 (152.3‐274.8)	0.393
ALT (U/L)	21.5 (14.0‐43.0)	23.5 (16.0‐42.3)	20.0 (14.0‐43.0)	0.516
AST (U/L)	26.0 (20.0‐40.5)	26.0 (19.5‐40.0)	26.5 (20.0‐43.3)	0.927
TBIL (umol/L)	9.9 (7.2‐13.3)	10.0 (7.2‐13.3)	9.7 (7.2‐13.3)	0.876
Albumin (g/L)	34.5 ± 0.4	34.9 ± 0.69	34.3 ± 0.44	0.475
Globulin (g/L)	31.2 (28.2‐35.8)	30.1 (27.7‐35.6)	32.2 (28.8‐36.0)	0.129
Cr (mmol/L)	74.0 (60.0‐93.0)	76.5 (66.5‐96.0)	70.5 (57.0‐93.0)	0.088
BUN (mmol/L)	5.4 (3.8‐8.3)	5.5 (3.6‐7.3)	5.3 (3.8‐8.4)	0.662
Uric acid (umol/L)	265.5 (197.2‐354.5)	266.4 (199.2‐421.8)	259.5 (194.6‐337.5)	0.240
TC (mmol/L)	3.5 (2.9‐4.1)	3.2 (2.7‐4.0)	3.6 (2.9‐4.2)	0.284
TG (mmol/L)	1.2 (1.0‐1.8)	1.1 (1.0‐1.8)	1.2 (1.0‐1.8)	0.441
HDL‐C (mmol/L)	0.9 (0.7‐1.1)	0.9 (0.7‐1.1)	0.9 (0.7‐1.2)	0.522
LDL‐C (mmol/L)	2.1 (1.6‐2.7)	1.9 (1.3‐2.6)	2.3 (1.6‐2.7)	0.045
K+ (mmol/L)	4.2 (3.8‐4.5)	4.0 (3.7‐4.4)	4.2 (3.8‐4.5)	0.164
Blood glucose (mmol/L)	6.1 (5.4‐7.8)	6.0 (5.4‐7.7)	6.2 (5.3‐8.1)	0.748
LDH (U/L)	264.0 (209.0‐392.0)	271.5 (201.0‐388.0)	264.0 (210.5‐395.0)	0.729
PT (s)	13.9 (13.3‐14.6)	13.5 (13.1‐14.4)	14.0 (13.3‐14.7)	0.050
APTT (s)	39.1 (36.3‐43.2)	38.9 (36.3‐43.7)	39.1 (36.1‐42.5)	0.956
D‐dimer (ug/ml)	0.97 (0.52‐2.39)	0.72 (0.40‐2.23)	1.09 (0.57‐2.45)	0.110
NT‐proBNP (pg/ml)	419.0 (125.0‐1330.0)	587.0 (130.5‐2932.5)	352 (124‐987.8)	0.325
hs‐cTnI (pg/ml)	8.9 (3.9‐33.2)	10.5 (5.2‐180.3)	8.0 (3.5‐23.8)	0.125
Mb (ug/L)	50.0 (30.7‐105.7)	63.0 (40.8‐117.3)	40.1 (28.7‐95.3)	0.033
CK (U/L)	64.0 (38.5‐143.5)	86.5 (43.5‐184.0)	59.0 (35.5‐127.5)	0.034
CK‐MB (U/L)	1.0 (0.6‐2.1)	1.4 (0.8‐2.8)	0.9 (0.5‐1.6)	0.013
IL‐6 (pg/ml)	12.6 (4.1‐37.4)	16.2 (4.6‐35.3)	11.6 (3.8‐37.4)	0.729
IL‐8 (pg/ml)	12.6 (7.4‐23.1)	11.5 (8.2‐20.4)	13.3 (7.3‐27.2)	0.510
TNF‐α (pg/ml)	9.0 (6.6‐11.8)	8.9 (6.8‐10.4)	9.2 (6.5‐12.2)	0.504
IL‐2R (U/ml)	655.0 (447.5‐993.8)	572.0 (446.0‐893.5)	676.0 (450.0‐1046.0)	0.217
hs‐CRP (pg/ml)	19.3 (2.9‐65.9)	20.5 (2.9‐60.8)	19.1 (2.9‐70.9)	0.946
Chest imaging				
Pleural lesions (%)	71 (38.8)	20 (38.5)	51 (38.9)	0.953
Ground‐glass opacity (%)	72 (39.3)	17 (3 2.7)	55 (42.0)	0.246
Patchy shadows (%)	125 (68.3)	37 (71.2)	88 (67.2)	0.602
Consolidation (%)	30 (16.4)	9 (17.3)	21 (16.0)	0.833
Pleural effusion (%)	33 (18.0)	12 (23.1)	21 (16.0)	0.263
Treatments				
High‐flow nasal cannula oxygen therapy (%)	150 (82.0)	44 (84.6)	106 (80.9)	0.557
Non‐invasive mechanical ventilation (%)	44 (24.0)	10 (19.2)	34 (26.0)	0.337
Invasive mechanical ventilation (%)	16 (8.7)	1 (1.9)	15 (11.5)	0.077
Outcomes				
Critically ill (%)	86 (47.0)	25 (48.1)	61 (46.6)	0.853
Mortality (%)	40 (21.9)	11 (21.2)	29 (22.1)	0.885

Abbreviation

COPD, chronic obstructive pulmonary disease; CKD, chronic kidney disease; DBP, diastolic blood pressure; SBP, systolic blood pressure; WBC, white blood cell; RBC, Red blood cell; Neut, neutrophil; Hb, haemoglobin; Lymph, lymphocyte; PLT, platelet; ALT, alanine aminotransferase; AST, aspartate aminotransferase; TBIL, Total Bilirubin; Cr, creatinine; BUN, blood urea nitrogen; TC, total cholesterol; TG, Triglyceride; HDL, high density lipoprotein; LDL, low‐density lipoprotein; K+, potassium; LDH, Lactate Dehydrogenase; PT, Prothrombin Time; APTT, activated partial thromboplastin time; hs‐cTnI, highly sensitive cardiac troponin I; Mb, myoglobin; CK, creatine kinase; CK‐MB, creatine kinase‐MB; IL6, interleukin 6; IL8, interleukin 8; TNF‐α, tumour necrosis factor‐α; IL‐2R, interleukin 2 receptor; hs‐CRP, highly sensitive C reaction protein; Categorical variables were presented as number (percentage) and normally and abnormally distributed continuous variables were represented by mean ±SD and median (first to third quartile, Q1‐Q3), respectively.

P values indicate differences between COVID‐19 patients with CAD treated with aspirin and without aspirin. *P* < .05 was considered statistically significant.

The 183 COVID‐19 hospitalized patients with CAD were further classified as aspirin group (n = 52) and non‐aspirin group (n = 131). As shown in Table 1, the mean age of the studied cohort was 71.2 years, and males made up about half. The total in‐hospital mortality of patients with CAD was 21.9%. There was no difference between the aspirin and non‐aspirin groups in age, sex, and pre‐existing comorbidities. Carefully analysing the laboratory results, there were no different in inflammatory cells count, liver function, renal function related examinations, some cytokines like IL6, IL8 and TNF‐α as well as pulmonary imaging performance between aspirin group and non‐Aspirin group. Besides, there were also no statistically significant differences in the rate of invasive mechanical ventilation and mortality between the two groups.

To rule out the influence of disease severity on the assessment of the relationship between aspirin and mortality, we further analysed critically severe COVID‐19 patients with CAD and divided them into critically severe‐aspirin group (mean age 71.3; male 60.0%) and critically severe‐non‐aspirin group (mean age 72.7; male 59.0%). The characteristics, laboratory results and clinical outcomes of these two groups were summarized in Table 2. There was no difference between critically severe‐aspirin group and critically severe‐non‐aspirin group in most laboratory examination findings as well as the mortality (44.0% vs. 45.9%; *P* = .872). These data suggested that the use of aspirin was not associated with the high risk of death in COVID‐19 patients with CAD in hospital.

**TABLE 2:** The characteristics and clinical outcomes of critically severe COVID‐19 patients with CAD

	COVID‐19 patients with CAD n = 86)	COVID‐19 patients with CAD treated with aspirin (n = 25)	COVID‐19 patients with CAD treated without aspirin (n = 61)	*P* value
Demographics and clinical characteristics				
Age (y)	72.3 ± 1.1	71.3 ± 1.8	72.7 ± 1.4	0.591
Sex				0.933
Male (%)	51 (59.3)	15 (60.0)	36 (59.0)	
Female (%)	35 (40.7)	10 (40.0)	25 (41.0)	
Comorbidity				
Hypertension (%)	48 (55.8)	17 (68.0)	31 (50.8)	0.145
Diabetes (%)	19 (22.1)	7 (28.0)	12 (19.7)	0.398
COPD (%)	3 (3.5)	1 (4.0)	2 (3.3)	1.00
CKD (%)	13 (15.1)	4 (16.0)	9 (14.8)	1.00
Cerebrovascular disease (%)	6 (7.0)	3 (12.0)	3 (4.9)	0.351
Symptoms and signs				
Fever (%)	56 (65.1)	15 (60.0)	41 (67.2)	0.524
Cough (%)	45 (52.3)	14 (56.0)	31 (50.8)	0.662
Dyspnoea (%)	33 (38.4)	8 (32.0)	25 (41.0)	0.437
Temperature (℃)	36.6 (36.3‐37.0)	36.5 (36.1‐37.0)	36.7 (36.3‐37.4)	0.058
Respiratory rate(/min)	22.0 (20.0‐25.0)	20.0 (19.0‐23.0)	22.0 (20.0‐30.0)	0.026
Heart rate (bpm)	92.0 (80.0‐104.0)	92.0 (80.0‐103.5)	91.5 (80.0‐107.3)	0.648
DBP (mmHg)	80.0 (70.0‐88.0)	80.0 (70.0‐95.5)	80.0 (72.0‐88.0)	0.716
SBP (mmHg)	132.5 ± 2.4	132.2 ± 4.7	132.6 ± 2.9	0.728
Laboratory findings				
WBC (*10^9/L)	7.8 (5.1‐11.1)	8.2 (5.9‐11.4)	7.7 (4.9‐10.9)	0.619
RBC (*10^9/L)	4.1 ± 0.1	4.0 ± 0.1	4.1 ± 0.1	0.839
Neut (*10^9/L)	6.2 (3.6‐8.9)	6.0 (4.3‐8.9)	6.5 (3.5‐9.0)	0.832
Hb (g/L)	122.0 (107.0‐138.0)	121.0 (100.5‐137.5)	122.5 (113.0‐138.5)	0.466
PLT (*10^9/L)	179.0 (138.5‐235.5)	207.0 (167.5‐293.0)	166.0 (125.5‐233.0)	0.016
ALT (U/L)	23.0 (14.0‐43.0)	25.0 (14.0‐44.0)	19.0 (14.0‐43.0)	0.563
AST (U/L)	33.0 (24.0‐46.0)	34.0 (25.0‐43.0)	32.5 (22.5‐49.0)	0.772
TBIL (umol/L)	10.4 (7.5‐15.7)	9.3 (6.5‐15.5)	10.9 (7.9‐16.4)	0.198
Albumin (g/L)	33.2 ± 0.5	34.4 ± 1.0	32.7 ± 0.6	0.234
Globulin (g/L)	33.2 (28.6‐37.6)	33.8 (28.2‐38.4)	33.2 (29.0‐37.4)	0.938
Cr (mmol/L)	83.0 (64.5‐108.5)	79.0 (72.0‐102.5)	83.5 (58.3‐121.5)	0.682
BUN (mmol/L)	6.7 (4.6‐11.1)	5.9 (5.0‐10.7)	6.9 (4.4‐11.1)	0.750
Uric acid (umol/L)	270.0 (183.2‐396.0)	266.9 (189.5‐495.4)	270.0 (179.9‐370.8)	0.579
TC (mmol/L)	3.4 (2.8‐4.0)	3.2 (2.6‐3.9)	3.5 (2.8‐4.0)	0.739
TG (mmol/L)	1.2 (1.0‐2.0)	1.1 (1.0‐1.9)	1.3 (1.0‐2.0)	0.317
HDL‐C (mmol/L)	0.9 (0.7‐1.0)	0.9 (0.7‐1.1)	0.8 (0.6‐1.0)	0.289
LDL‐C (mmol/L)	2.0 (1.5‐2.6)	2.0 (1.3‐2.7)	2.0 (1.6‐2.6)	0.707
K+ (mmol/L)	4.3 (3.9‐4.6)	4.2 (3.9‐4.6)	4.4 (3.8‐4.7)	0.437
Blood Glucose (mmol/L)	6.6 (5.5‐9.4)	6.9 (5.6‐11.5)	6.5 (5.4‐8.9)	0.355
LDH (U/L)	340.5 (242.0‐525.3)	335.0 (246.5‐506.0)	349.0 (232.0‐532.0)	0.934
PT (s)	14.1 (13.4‐15.6)	14.0 (13.2‐15.5)	14.3 (13.6‐15.8)	0.083
APTT (s)	40.5 (37.2‐44.0)	39.1 (37.3‐43.5)	40.9 (37.1‐45.4)	0.400
D‐dimer (ug/ml)	1.7 (0.8‐3.1)	1.0 (0.7‐3.2)	1.7 (0.8‐3.2)	0.560
NT‐proBNP (pg/ml)	1165.0 (221.0‐5638.0)	3397.0 (1640.0‐11134.0)	725.0 (183.0‐1790.0)	0.007
hs‐cTnI (pg/ml)	23.4 (8.2‐210.9)	74.8 (12.4‐1100.4)	16.8 (7.4‐96.1)	0.070
Mb (ug/L)	92.4 (40.9‐223.9)	99.0 (51.4‐202.0)	87.3 (35.8‐242.5)	0.458
CK (U/L)	83.0 (41.0‐265.0)	130.0 (55.5‐234.5)	73.0 (34.5‐265.5)	0.212
CK‐MB (U/L)	1.4 (0.7‐3.0)	2.35 (1.2‐3.9)	1.2 (0.7‐2.6)	0.076
IL‐6 (pg/ml)	33.7 (13.0‐68.0)	28.9 (15.2‐54.1)	35.6 (13.0‐89.1)	0.388
IL‐8 (pg/ml)	16.9 (10.6‐33.2)	11.8 (7.7‐20.2)	22.3 (11.8‐39.2)	0.012
TNF‐α (pg/ml)	10.3 (8.0‐13.4)	9.0 (7.9‐12.1)	11.5 (8.0‐13.7)	0.151
IL‐2R (U/ml)	808.0 (539.5‐1155.0)	622.0 (466.0‐988.0)	935.0 (671.8‐1237.0)	0.019
hs‐CRP (pg/ml)	51.9 (11.9‐110.9)	38.4 (11.8‐80.6)	60.6 (11.6‐118.1)	0.371
Chest imaging				
Pleural lesions (%)	29 (33.7)	8 (32.0)	21 (34.4)	0.829
Ground‐glass opacity (%)	25 (29.1)	5 (20.0)	20 (32.8)	0.236
Patchy shadows (%)	45 (52.3)	15 (60.0)	30 (49.2)	0.362
Consolidation (%)	10 (11.6)	2 (8.0)	8 (13.1)	0.763
Pleural effusion (%)	18 (20.9)	8 (32.0)	10 (16.4)	0.106
Treatments				
High‐flow nasal cannula oxygen therapy (%)	79 (91.9)	25 (100.0)	54 (88.5)	0.101
Non‐invasive mechanical ventilation (%)	41 (47.7)	10 (40.0)	31 (50.8)	0.362
Invasive mechanical ventilation (%)	15 (17.4)	1 (4.0)	14 (23.0)	0.073
Outcomes				
Mortality (%)	39 (45.3)	11 (44.0)	28 (45.9)	0.872

Abbreviation

COPD, chronic obstructive pulmonary disease; CKD, chronic kidney disease; DBP, diastolic blood pressure; SBP, systolic blood pressure; WBC, white blood cell; RBC, Red blood cell; Neut, neutrophil; Hb, haemoglobin; Lymph, lymphocyte; PLT, platelet; ALT, alanine aminotransferase; AST, aspartate aminotransferase; TBIL, Total Bilirubin; Cr, creatinine; BUN, blood urea nitrogen; TC, total cholesterol; TG, Triglyceride; HDL, high density lipoprotein; LDL, low‐density lipoprotein; K+, potassium; LDH, Lactate Dehydrogenase; PT, Prothrombin Time; APTT, activated partial thromboplastin time; hs‐cTnI, highly sensitive cardiac troponin I; Mb, myoglobin; CK, creatine kinase; CK‐MB, creatine kinase‐MB; IL6, interleukin 6; IL8, interleukin 8; TNF‐α, tumour necrosis factor‐α; IL‐2R, interleukin 2 receptor; hs‐CRP, highly sensitive C reaction protein; Categorical variables were presented as number (percentage) and normally and abnormally distributed continuous variables were represented by mean ± SD and median (first to third quartile, Q1‐Q3), respectively

P values indicate differences between critically severe COVID‐19 patients with CAD treated with aspirin and without aspirin. *P* < .05 was considered statistically significant.

To investigate the major risk factors for death of COVID‐19 patients with CHD. We further divided these 183 patients into two groups according to their final clinical outcome (survive or die). The clinical characteristics and treatments between survivors and non‐survivors were analysed and summarized in Table 3. Our results shown that the clinical characteristics of patients from non‐survivor group were much worse than survivor group, manifested as older in age, higher proportion of male, more severe inflammation, and organ damage (including myocardial injury). In addition, multivariate logistic regression model analyses were performed for identifying independent correlative factor of mortality of COVID‐19 patients with CAD. The results exhibited that the age, proportion of male and the existing complication of CKD were positively correlated with mortality, the usage of low‐dose aspirin was not correlated with mortality (Table 4).

**TABLE 3:** The characteristics and clinical outcomes of survivor and non‐survivor COVID‐19 patients with CAD

	COVID‐19 patients with CAD			*P* value
	Total (n = 183)	Survivor (n = 143)	Non‐survivor (n = 40)	
Demographics and clinical characteristics				
Age (y)	71.2 ± 0.7	70.5 ± 0.8	73.9 ± 1.6	0.041
Male (%)	99 (54.1)	70 (49.0)	29 (72.5)	0.008
Comorbidity				
Hypertension (%)	102 (55.7)	78 (54.5)	24 (60.0)	0.539
Diabetes (%)	40 (21.9)	32 (22.4)	8 (20.0)	0.748
COPD (%)	8 (4.4)	7 (4.9)	1 (2.5)	0.828
CKD (%)	18 (9.8)	9 (6.3)	9 (22.5)	0.006
Cerebrovascular disease (%)	9 (4.9)	7 (4.9)	2 (5.0)	1.00
Symptoms and signs				
Temperature (℃)	36.5 (36.3‐37.0)	36.5 (36.3‐37.0)	36.8 (36.2‐37.3)	0.374
Fever (%)	127 (69.4)	99 (69.2)	28 (70.0)	0.926
Cough (%)	105 (57.4)	84 (58.7)	21 (52.5)	0.480
Dyspnoea (%)	73 (39.9)	58 (40.6)	15 (37.5)	0.727
Respiratory rate (/min)	20.0 (20.0‐25.0)	20.0 (19.0‐24.0)	24.0 (20.0‐32.0)	0.002
Heart rate (bpm)	88.0 (78.0‐100.0)	88.0 (78.0‐99.0)	90.0 (78.0‐103.0)	0.271
DBP (mmHg)	80.0 (73.0‐88.0)	80.0 (73.0‐88.0)	80.0 (70.0‐88.5)	0.896
SBP (mmHg)	133.8 ± 1.4	135.2 ± 1.5	129.0 ± 3.6	0.096
Laboratory findings				
WBC (*10^9/L)	6.1 (5.0‐8.5)	5.7 (4.6‐7.4)	9.0 (7.1‐16.4)	<0.001
RBC (*10^12/L)	4.01 ± 0.05	3.96 ± 0.05	4.20 ± 0.13	0.246
Neut (*10^9/L)	4.2 (3.0‐6.8)	3.8 (2.9‐5.4)	7.5 (5.4‐15.4)	<0.001
Lymph (*10^9/L)	1.0 (0.7‐1.5)	1.2 (0.8‐1.6)	0.6 (0.5‐0.8)	<0.001
Hb (g/L)	123.0 (110.0‐136.0)	123.0 (110.0‐134.0)	128.0 (115.0‐150.0)	0.071
PLT (*10^9/L)	209.0 (152.8‐285.0)	223.0 (159.0‐296.0)	163.0 (120.0‐219.0)	0.001
ALT (U/L)	21.5 (14.0‐43.0)	20.0 (14.0‐38.0)	26.0 (18.0‐43.0)	0.042
AST (U/L)	26.0 (20.0‐40.5)	25.0 (19.0‐35.0)	40.0 (25.0‐58.0)	<0.001
TBIL (umol/L)	9.9 (7.2‐13.3)	9.2 (7.1‐12.1)	13.4 (9.3‐18.6)	<0.001
Albumin (g/L)	34.5 ± 0.4	35.4 ± 0.4	31.4 ± 0.8	<0.001
Globulin (g/L)	31.2 (28.2‐35.8)	30.5 (27.6‐34.3)	36.7 (30.5‐38.6)	<0.001
Cr (mmol/L)	74.0 (60.0‐93.0)	68.0 (57.0‐86.0)	96.0 (75.0‐127.0)	<0.001
BUN (mmol/L)	5.4 (3.8‐8.3)	4.9 (3.6‐6.6)	9.7 (6.5‐12.8)	<0.001
Uric acid (umol/L)	265.5 (197.2‐354.5)	259.0 (197.9‐347.3)	286.0 (193.2‐462.0)	0.149
TC (mmol/L)	3.5 (2.9‐4.1)	3.6 (2.9‐4.2)	3.1 (2.7‐4.0)	0.104
TG (mmol/L)	1.2 (1.0‐1.8)	1.2 (1.0‐1.7)	1.4 (1.0‐2.0)	0.039
HDL‐C (mmol/L)	0.9 (0.7‐1.1)	0.9 (0.8‐1.2)	0.9 (0.6‐0.9)	0.006
LDL‐C (mmol/L)	2.1 (1.6‐2.7)	2.1 (1.6‐2.7)	1.8 (1.2‐2.5)	0.278
K+ (mmol/L)	4.2 (3.8‐4.5)	4.1 (3.8‐4.5)	4.4 (3.8‐4.8)	0.048
Blood Glucose (mmol/L)	6.1 (5.4‐7.8)	5.9 (5.2‐6.9)	7.8 (6.4‐11.1)	<0.001
LDH (U/L)	264.0 (209.0‐392.0)	246.0 (201.0‐316.0)	486.0 (316.3‐666.0)	<0.001
PT (s)	13.9 (13.3‐14.6)	13.6 (13.2‐14.3)	15.1 (14.0‐16.9)	<0.001
APTT (s)	39.1 (36.3‐43.2)	38.8 (35.6‐42.5)	29.8 (37.0‐45.3)	0.088
D‐dimer (ug/ml)	0.97 (0.52‐2.39)	0.82 (0.45‐1.68)	3.32 (0.92‐8.12)	<0.001
NT‐proBNP (pg/ml)	125.0 (419.0‐1330.0)	233.0 (88.5‐605.0)	1940.5 (1092.5‐8964.0)	<0.001
hs‐cTnI (pg/ml)	8.9 (3.9‐33.2)	6.8 (2.8‐13.7)	74.8 (11.0‐800.3)	<0.001
Mb (ug/L)	50.0 (30.7‐105.7)	40.5 (28.7‐71.1)	144.6 (95.6‐291.2)	<0.001
CK (U/L)	64.0 (38.5‐143.5)	57.0 (37.0‐97.0)	196.5 (57.3‐423.5)	<0.001
CK‐MB (U/L)	1.0 (0.6‐2.1)	0.9 (0.5‐1.6)	2.4 (1.1‐6.3)	<0.001
IL‐6 (pg/ml)	12.6 (4.1‐37.4)	8.1 (3.4‐20.6)	59.0 (35.6‐169.8)	<0.001
IL‐8 (pg/ml)	12.6 (7.4‐23.1)	11.4 (6.7‐20.4)	22.7 (11.8‐40.8)	<0.001
TNF‐α (pg/ml)	9.0 (6.6‐11.8)	8.9 (6.3‐11.1)	9.9 (7.0‐13.4)	0.038
IL‐2R (U/ml)	655.0 (447.5‐993.8)	594.0 (402.0‐874.0)	993.0 (717.0‐1480.0)	<0.001
hs‐CRP (pg/ml)	19.3 (2.9‐65.9)	11.1 (2.5‐41.9)	100.7 (44.0‐156.6)	<0.001
Chest imaging				
Pleural lesions (%)	71 (38.8)	66 (46.2)	5 (12.5)	<0.001
Ground‐glass opacity (%)	72 (39.3)	71 (49.7)	1 (2.5)	<0.001
Patchy shadows (%)	125 (68.3)	119 (83.2)	6 (15.0)	<0.001
Consolidation (%)	30 (16.4)	29 (20.3)	1 (2.5)	0.007
Pleural effusion (%)	33 (18.0)	31 (21.7)	2 (5.0)	0.015
Treatments				
High‐flow nasal cannula oxygen therapy	150 (82.0)	112 (78.3)	38 (95.0)	0.015
Non‐invasive mechanical ventilation	44 (24.0)	14 (9.8)	30 (75.0)	<0.001
Invasive mechanical ventilation	16 (8.7)	3 (2.1)	13 (32.5)	<0.001
**Aspirin usage (%)**	52 (28.4)	41 (28.7)	11 (27.5)	0.885

Abbreviations

COPD, chronic obstructive pulmonary disease; CKD, chronic kidney disease; DBP, diastolic blood pressure; SBP, systolic blood pressure; WBC, white blood cell; RBC, Red blood cell; Neut, neutrophil; Hb, haemoglobin; Lymph, lymphocyte; PLT, platelet; ALT, alanine aminotransferase; AST, aspartate aminotransferase; TBIL, Total Bilirubin; Cr, creatinine; BUN, blood urea nitrogen; TC, total cholesterol; TG, Triglyceride; HDL, high density lipoprotein; LDL, low‐density lipoprotein; K+, potassium; LDH, Lactate Dehydrogenase; PT, Prothrombin Time; APTT, activated partial thromboplastin time; hs‐cTnI, highly sensitive cardiac troponin I; Mb, myoglobin; CK, creatine kinase; CK‐MB, creatine kinase‐MB; IL6, interleukin 6; IL8, interleukin 8; TNF‐α, tumour necrosis factor‐α; IL‐2R, interleukin 2 receptor; hs‐CRP, highly sensitive C reaction protein; Categorical variables were presented as number (percentage) and normally and abnormally distributed continuous variables were represented by mean ± SD and median (first to third quartile, Q1‐Q3), respectively

P values indicate differences between survivors and non‐survivors. *P* < .05 was considered statistically significant.

**TABLE 4:** Multivariate correlative factors of mortality in COVID‐19 with CAD

	Multivariate analysis	
	OR (95% CI)	p value
Age	1.041 (1.001‐1.083)	0.046
Sex ratio	2.677 (1.205‐5.950)	0.016
Treating with aspirin	0.944 (0.411‐2.172)	0.893
CKD	3.335 (1.176‐9.463)	0.024

Abbreviation

CKD, chronic kidney disease.

## 4 DISCUSSION

The report on March 21, 2020 showed that the mortality of COVID‐19 in China was 4.0%, while our data for mortality was 9.6%, mainly because of the patients enrolled in the present study were from Tongji hospital, where admitted more critically ill COVID‐19 patients in Wuhan.^14^ Besides, the case fatality rate rises rapidly with increasing age or medical comorbidities.^15^ Previous research reported that the mortality of COVID‐19 patients with cardiovascular disease (CVD) was as much as 10.5%, while our cohort showed that the mortality of patients with coronary artery disease was 21.9%, which was much higher than patients with hypertension, diabetes or COPD.^15^ It was reported that the prevalence of CVD in SARS and MERS was 8% and 30% respectively, while varied from 8% to 15% in COVID‐19.^2^, ^3^ CAD was present in approximately 6.3% of patients in our study which was similar to other literature between 2.5% and 17%.^4^, ^16^ Possible explanations for the higher incidence in COVID‐19 patients with CAD include elderly, decreased immune system function, increased ACE2, and predisposition.^17^

Low‐dose aspirin is an most widely used agents for the secondary prevention of cardiovascular disease, and had been proved to effectively reduce the rates of both myocardial infarction and ischaemic stroke.^5^, ^6^, ^18^, ^19^, ^20^, ^21^ Viral infection was an unstable risk factor for chronic cardiovascular disease. The reduction of intrinsic heart reserve due to chronic cardiovascular disease was further imbalanced by the increased metabolic demands caused by viral infection. Existing evidence indicated that novel SARS‐CoV‐2 can significantly increase the risk of acute cardiovascular events in patients with coronary artery disease and heart failure by causing systemic inflammatory reactions, and destabilizing coronary artery plaques. while pneumonia may further directly or indirectly affect the cardiovascular system.^17^ However, there is an international debate among healthcare professionals regarding the usage of NSAIDs in COVID‐19 patients^22^ for that NSAIDs may alter the function of neutrophils, delay bacterial clearance and inflammation resolution, and they are associated with the development of pleuropulmonary complications (pleural abscess, excavation, and abscess). In addition, thrombocytopenia, caused by decreased production in infected hematopoietic marrow as well as increased consumption by DIC, result in damaged lung tissue and capillaries is present in 5%‐41.7% COVID‐19 patients.^9^, ^23^ Aspirin irreversibly inhibits platelet cyclooxygenase, and its effect could persist for the circulating life of platelets (7‐10 days). Therefore, using of aspirin may increase the risk of bleeding in COVID‐19 patients by severe thrombocytopenia. Furthermore, although glucocorticoid is not recommended by the world health organization (WHO), it has been reported that 44.9% of COVID‐19 patients were treated with glucocorticoid, which further increased the risk of aspirin‐induced bleeding. Similarly, the prophylactic use of low‐dose aspirin in COVID‐19 infected pregnant women at risk of placental complications is controversial.^24^, ^25^ The WHO has issued an official statement recommending not to avoid the use of Ibuprofen based on the current available data.^26^ To our knowledge, there is limited data to suggest the association between prophylactic use of low‐dose aspirin and progression of COVID‐19 infection. Our data suggested that the usage of low‐dose aspirin was not associated with the mortality. Although clinical benefit from low‐dose aspirin were not seen in our data, we still recommend that COVID‐19 patients with coronary artery disease should not stop taking aspirin unless under some special conditions.

ACC also issued guidance on cardiac implications of coronavirus on February 14, 2020. This guidance suggested that the rigorous use of plaque‐stabilizing agents (statins, beta‐blockers, ACE inhibitors, acetylsalicylic acid) could offer additional protection to CVD patients during COVID‐19 epidemic.^27^

Furthermore, it is indeed an important issue to evaluate the relationship between the use of low‐dose aspirin and thrombosis in COVID‐19 patients. Many studies have shown that COVID‐19 patients had coagulation dysfunction and a high incidence of thromboembolic events.^28^, ^29^ Low‐dose aspirin was widely utilized for prevention of stroke and myocardial infarction in high‐risk patients because of its antiplatelet properties, so it might reduce the incidence of COVID‐19 induced coagulopathy and prevent severe deterioration due to cardiovascular complications. Based on our data, there is no difference of hs‐cTnI, a sensitive indicator of myocardial infarction, between aspirin group [10.5 pg/ml, IQR (5.2‐180.3)] and non‐aspirin group [8.0pg/ml, IQR (3.5‐23.8)]. Similar with our results, another observational study composed of 412 COVID‐19 patients reported that aspirin had no effect on the rate of overt thrombosis.^30^ In fact, coagulation dysfunction in COVID‐19 patients is mainly manifested as thrombocytopenia, prolonged prothrombin time and elevated D‐Dimer, which are indicators of DIC. Studies have shown that patients with COVID‐19 associated coagulopathy who received prophylactic heparin had a lower mortality than patients not receiving anticoagulant treatment.^31^ Interestingly, our data showed that D‐Dimer was significantly higher in COVID‐19 patients with CAD than patients without CAD. However, the use of low‐dose aspirin had no effect on the elevated D‐Dimer in patients with CAD. In general, randomized controlled trials are needed to assess whether aspirin has the protective effect in COVID‐19 patients with coronary heart disease.

Overall, our data indicated that pre‐hospital use of low‐dose aspirin was not associated with high in‐hospital mortality in COVID‐19 patients with coronary artery disease. Furthermore, cardiovascular care team should pay more attention to elderly male patients with CAD for their high mortality of COVID‐19. For covid‐19 positive patients, the risk/benefit of low‐dose aspirin therapy should be fully evaluated, including indications, age, and platelet counts, before discontinuation of aspirin.

### 4.1 Limitations

This study has several limitations. First, this is a retrospective study, so it may be biased by differences in patients taking vs. not taking aspirin at the time of hospitalization. Second, the study sample‐size was not big enough for fully clarified effect of low‐dose aspirin using in COVID‐19 patient. Third, the current findings may not be generalizable to all patients with coronary artery disease. Because the study was based on inpatients, it was difficult to reflect the effect of aspirin of outpatient setting or ethnically or geographically diverse populations. Therefore, international large‐scale prospective cohort studies and randomized controlled trials are needed to better determine the association between low‐dose aspirin and survival in COVID‐19.

## 5 CONCLUSIONS

Among patients with coronary artery disease hospitalized by COVID‐19, pre‐hospitalization treatment with low‐dose aspirin was not associated with all‐cause mortality of COVID‐19 patients with CHD. Despite the possibility of residual confounders, we do not recommend immediate cessation of aspirin in hospitalized patients.

## CONFLICT OF INTEREST

The authors declare that they have no competing financial interests or personal relationships that could influence the work reported in this paper.

## Supporting information

Table S1
