# Supplementary material for: Mortality and pre‐hospitalization use of low‐dose aspirin in COVID‐19 patients with coronary artery disease
Source: J Cell Mol Med. 2020 Dec 18;25(2):1263–73. doi: 10.1111/jcmm.16198 (PMC7812246; doi:10.1111/jcmm.16198)
Supplement: Supplementary file 1 — Table S1 [file JCMM-25-1263-s001.docx]

**Supplemental Table 1. The characteristics and clinical outcomes of COVID-19 patients with or without CAD.**

|  | **COVID-19 patients**  **(n=2886)** | **COVID-19 patients without CAD**  **(n=2703)** | **COVID-19 patients with CAD**  **(n=183)** | **p value** |
| --- | --- | --- | --- | --- |
| **Demographics and clinical characteristics** | | | | |
| Age (y) | 59.1 ± 0.3 | 58.3 ± 0.3 | 71.2 ± 0.7 | <0.001 |
| Male (%) | 1402 (49.1) | 1326 (49.1) | 99 (54.1） | 0.701 |
| Comorbidity |  |  |  |  |
| Hypertension (%) | 717 (25.1) | 628 (23.2) | 102 (55.7) | <0.01 |
| Diabetes (%) | 361 (12.7) | 319 (11.8) | 40 (21.9) | <0.01 |
| COPD (%) | 35 (1.2) | 30 (1.1) | 8 (4.4) | 0.043 |
| CKD (%) | 16 (0.6) | 12 (0.4) | 18 (9.8) | 0.008 |
| Cerebrovascular disease (%) | 90 (3.2) | 81 (3.0) | 9 (4.9) | 0.071 |
| Respiratory rate (/min) | 20.0 (20.0-22.0) | 20.0 (20.0-22.0) | 20.0 (20.0-25.0) | 0.044 |
| Heart rate (bpm) | 90.0 (80.0-102.0) | 90.0 (80.0-102.0) | 88.0 (78.0-100.0) | 0.217 |
| DBP (mmHg) | 80.0 (72.0-89.0) | 80.0 (72.0-89.0) | 80.0 (73.0-88.0) | 0.731 |
| SBP (mmHg) | 175.5 ± 44.9 | 178.4 ± 48.0 | 133.8 ± 1.4 | 0.014 |
| **Laboratory findings** | | | | |
| WBC (*10^9/L) | 5.8 (4.6-7.6) | 5.8 (4.6-7.5) | 6.08 (4.99-8.46) | 0.003 |
| RBC (*10^12/L) | 4.15 ± 0.01 | 4.16 ± 0.01 | 4.01 ± 0.05 | 0.001 |
| Neut (*10^9/L) | 3.8 (2.7-5.4) | 3.8 (2.7-5.4) | 4.16 (3.05-6.79) | <0.001 |
| Hb (g/L) | 127.0 (116.0-139.0) | 128.0 (116.0-139.0) | 123.0 (110.0-136.0) | 0.010 |
| PLT (*10^9/L) | 219.0 (166.0-282.0) | 220.0 (167.0-282.0) | 209.0 (152.8-285.0) | 0.078 |
| ALT (U/L) | 22.0 (14.0-37.0) | 22.0 (14.0-37.0) | 21.5 (14.0-43.0) | 0.832 |
| AST (U/L) | 25.0 (18.0-36.0) | 25.0 (18.0-36.0) | 26.0 (20.0-40.5) | 0.010 |
| TBIL (umol/L) | 8.9 (6.5-12.1) | 8.8 (6.4-12.1) | 9.9 (7.2-13.3) | 0.001 |
| Albumin (g/L) | 36.4 ± 0.1 | 36.5 ± 0.1 | 34.5 ± 0.4 | <0.001 |
| Globulin (g/L) | 31.8 (28.3-35.6) | 31.8 (28.3-35.6) | 31.2 (28.2-35.8) | 0.897 |
| Cr (mmol/L) | 68.0 (56.0-83.0) | 67.0 (56.0-82.0) | 74.0 (60.0-93.0) | <0.001 |
| BUN (mmol/L) | 4.5 (3.5-5.8) | 4.4 (3.4-5.7) | 5.4 (3.8-8.3) | <0.001 |
| Uric acid (umol/L) | 260.0 (201.1-332.0) | 260.0 (202.0-331.6) | 265.5 (197.2-354.5) | 0.508 |
| TC (mmol/L) | 3.8 (3.2-4.5) | 3.8 (3.3-4.5) | 3.5 (2.9-4.1) | <0.001 |
| TG (mmol/L) | 1.3 (1.0-1.9) | 1.3 (1.0-1.9) | 1.2 (1.0-1.8) | 0.048 |
| HDL-C (mmol/L) | 1.0 (0.8-1.2) | 1.0 (0.8-1.2) | 0.9 (0.7-1.1) | 0.018 |
| LDL-C (mmol/L) | 2.5 (1.9-3.0) | 2.5 (2.0-3.1) | 2.1 (1.6-2.7) | <0.001 |
| K+ (mmol/L) | 4.2 (3.9-4.5) | 4.2 (3.9-4.5) | 4.15 (3.77-4.52) | 0.424 |
| Blood glucose (mmol/L) | 5.9 (5.1-7.4) | 5.9 (5.1-7.4) | 6.1 (5.4-7.8) | 0.013 |
| LDH (U/L) | 246.0 (193.0-331.0) | 244.0 (192.0-329.0) | 264.0 (209.0-392.0) | 0.001 |
| PT (s) | 13.7 (13.2-14.4) | 13.7 (13.2-14.4) | 13.9 (13.3-14.6) | 0.056 |
| APTT (s) | 38.8 (35.9-42.4) | 38.7 (35.9-42.4) | 39.1 (36.3-43.2) | 0.416 |
| D-dimer (ug/ml) | 0.7 (0.3-1.6) | 0.7 (0.3-1.6) | 0.97 (0.52-2.39) | <0.001 |
| NT-proBNP (pg/ml) | 119.0 (45.0-440.8） | 115.0 (44.0-410.0) | 419.0 (125.0-1330.0) | <0.001 |
| hs-cTnI (pg/ml) | 6.6 (3.5-17.3) | 6.4 (3.4-17.0) | 8.9 (3.9-33.2) | 0.057 |
| Mb (ug/L) | 36.2 (26.2-63.0) | 35.6 (25.9-61.1) | 50.0 (30.7-105.7) | <0.001 |
| CK (U/L) | 61.0 (40.0-101.0) | 61.0 (40.0-99.0) | 64.0 (38.5-143.5) | 0.63 |
| CK-MB (U/L) | 0.7 (0.4-1.2) | 0.7 (0.4-1.1) | 1.0 (0.6-2.1) | <0.001 |
| IL-6 (pg/ml) | 7.5 (3.1-26.0) | 7.2 (3.1-25.2) | 12.6 (4.1-37.4) | 0.003 |
| IL-8 (pg/ml) | 12.0 (7.9-22.2) | 11.9 (7.9-22.0) | 12.6 (7.4-23.1） | 0.954 |
| TNF-α (pg/ml) | 8.1 (6.4-10.7) | 8.1 (6.4-10.6) | 9.0 (6.6-11.8) | 0.072 |
| IL-2R (U/ml) | 513.0 (324.0-800.5) | 503.0 (316.0-791.0) | 655.0 (447.5-993.8) | <0.001 |
| hs-CRP (pg/ml) | 11.8 (1.7-58.9) | 11.2 (1.7-57.9) | 19.3 (2.9-65.9) | 0.005 |
| **Treatments** | | | | |
| High-flow nasal cannula oxygen therapy | 1911 (67.0) | 1789 (66.2) | 150 (82.0) | <0.001 |
| Non-invasive mechanical ventilation | 325 (11.4) | 297 (11.0) | 44 (24.0) | 0.004 |
| Invasive mechanical ventilation | 104 (3.6) | 94 (3.5) | 16 (8.7) | 0.043 |
| **Outcomes** | | | | |
| Mortality (%) | 274 (9.6) | 246 (9.1) | 40 (21.9) | <0.001 |

COPD, chronic obstructive pulmonary disease; CKD, chronic kidney disease; DBP, diastolic blood pressure; SBP, systolic blood pressure; WBC, white blood cell; RBC, Red blood cell; Neut, neutrophil; Hb, hemoglobin; Lymph, lymphocyte; PLT, platelet; ALT, alanine aminotransferase; AST, aspartate aminotransferase; TBIL, Total Bilirubin; Cr, creatinine; BUN, blood urea nitrogen; TC, total cholesterol; TG, Triglyceride; HDL, high density lipoprotein; LDL, low-density lipoprotein; K+, potassium; LDH, Lactate Dehydrogenase; PT, Prothrombin Time; APTT, activated partial thromboplastin time; hs-cTnI, highly sensitive cardiac troponin I; Mb, myoglobin; CK, creatine kinase; CK-MB, creatine kinase-MB; IL6, interleukin 6; IL8, interleukin 8; TNF-α, tumor necrosis factor-α; IL-2R, interleukin 2 receptor; hs-CRP, highly sensitive C reaction protein; Categorical variables were presented as number (percentage) and continuous variables were presented as median (first to third quartile, Q1-Q3).

P values indicate differences between COVID-19 patients with CAD and COVID-19 patients without CAD. P < 0.05 was considered statistically significant.
